# Spatial Heterogeneity of Social Vulnerabilities Associated with Colorectal Cancer Screening Rates in California

**DOI:** 10.1093/geroni/igaf122.4011

**Published:** 2025-12-31

**Authors:** Jinbo Niu, Joonhyeog Park, Tamara Cadet

**Affiliations:** University of Southern California, Los Angeles, California, United States; University of Pennsylvania, Philadelphia, Pennsylvania, United States; University of Pennsylvania, Philadelphia, Pennsylvania, United States

## Abstract

Colorectal cancer (CRC) is the second leading cause of cancer-related deaths in the United States, and the American Cancer Society recommends CRC screening beginning at age 45. Despite its economic prosperity, California in 2022 reported the lowest CRC screening rates among adults aged 45 to 75 in the U.S. This counterintuitive phenomenon raises concerns about which social vulnerability factors are associated with the low rate in California. However, prior research on social vulnerability has largely focused on national-level patterns, with limited attention to California-specific disparities and the spatial heterogeneity of social vulnerability factors associated with CRC screening rates. This study aimed to explore the spatial heterogeneity of social vulnerabilities influencing CRC screening rates in California by analyzing the California tract-level CRC screening rate from the CDC PLACES data and the total 16 social vulnerability variables from the CDC Social Vulnerability Index (SVI) data in 2022. The most associated SVI factors were selected through stepwise regression, with their spatially varying effects examined via Geographically Weighted Regression. Among the top five associated SVI factors selected by stepwise regression, educational attainment and limited English proficiency showed the greatest spatial heterogeneity, with effects ranging from strongly negative to positive across regions. The percentage of adults over 65 maintained an overall positive association with screening rates but with little spatial variation. These findings highlight the need for spatially adaptive language and education interventions that address local vulnerabilities. Meanwhile, prioritizing CRC screening among adults aged 45–65 is essential to narrowing screening gaps in California.

